# Overexpression of Inducible Nitric Oxide Synthase in Allergic and Nonallergic Nasal Polyp

**DOI:** 10.1155/2019/7506103

**Published:** 2019-11-07

**Authors:** Ahmed Adel Sadek, Soha Abdelwahab, Safaa Yehia Eid, Riyad A. Almaimani, Mohammad A. Althubiti, Mahmoud Zaki El-Readi

**Affiliations:** ^1^Department of Otorhinolaryngology, Faculty of Medicine, Minia University, Minia 61511, Egypt; ^2^Department of Histology, Faculty of Medicine, Minia University, Minia 61511, Egypt; ^3^Department of Biochemistry, Faculty of Medicine, Umm Al-Qura University, Makkah, Saudi Arabia; ^4^Department of Biochemistry, Faculty of Pharmacy, Al-Azhar University, 71524 Assiut, Egypt

## Abstract

Sinonasal polyps are very common benign lesions of the nasal mucosa. Most of nasal polyps (NP) are idiopathic, and the pathophysiology of this disease is still incompletely understood. Nitric oxide (NO) is a reactive molecule generated by nitric oxide synthase (NOS). NO has been identified as an important mediator in airway function and pathogenesis of several respiratory system diseases. Histological and genetical expression of iNOS was detected to evaluate the role of NO in the pathogenesis of allergic (ANP) and nonallergic nasal polyps (NANP). Forty patients with nasal polyps (20 allergic and 20 nonallergic) were identified by history, clinical examination, and investigation. NPs were obtained from the middle turbinate (MT) during concha bullosa surgery. Twenty normal MT nasal tissues were taken as the control from patients undergoing concha bullosa surgery, without any evidence of allergy or inflammation. A nasal polyp specimen from each patient was subjected for immune-histochemical study followed by histological examination to detect the expression of iNOS. RT-PCR was used to evaluate the iNOS gene expression in isolated tissues. The expression of iNOS in both epithelial and stromal layers was greater in NP than in MT tissues. The ANP group showed more iNOS expression than those of the NANP group. The relative mRNA levels of iNOS gene were significantly higher in ANP (2.5-fold) compared to the normal (1.02-fold, *P* < 0.001) and NANP (1.5-fold, *P* < 0.01) groups. NP exhibited a significantly high expression of iNOS at both histological and genetical levels. NO might be an essential factor in the life history of NP. Further studies in a larger sample size are required to explain the probable mechanisms of NO in pathogenesis of NP.

## 1. Introduction

Nasal polyps (NPs) have been stated to happen in 0.5%-4% of the population, with the presence of popular asymptomatic NPs in elder patients [1]. NPs commonly initiate from the ethmoid cells that comprise an epithelial inside layer adjacent primary edema, glandular hyperplasia, fibrosis, and eosinophilic infiltration. These are the distinguishing histological characters of NPs. The polypoid stroma is extremely edematous with a fluctuating mass of inflammatory cells. However, the underlying pathophysiology of NPs is left not well understood. They have long been accompanying rhinitis, acetyl salicylic acid (aspirin) sensitivity, cystic fibrosis, Kartagener's syndrome, and bronchial asthma [2]. Nevertheless, the role of allergy in the history of the disease and pathogenesis of NPs is debatable [2]. Nitric oxide (NO) has been known as a vital mediator in several physiological and inflammatory conditions [3]. NO is produced from 3 isoforms of NOS: NOS1 (neuronal, nNOS), NOS2 (inducible, iNOS), and NOS3 (endothelial, eNOS). In the human air route, mostly the nasal mucosa, all 3 isoforms of NOS are initiated in epithelial cells [4].

Scientific interest is intensive on the impact of NO in the respiratory system, especially airway function. NPs constitute a chronic inflammatory process, and NO plays an important role, both in acute and chronic inflammations [5].

It has been proposed that NO must be measured to know the pathogenesis of NP [6]. It has been reported that the activity of total NOS was increased in NPs than normal nasal mucosa. There are some studies suggesting that NOS is highly expressed in NPs than normal [7–11]. According to our information, there is a small number of evidences to evaluate potential differences between allergic and nonallergic nasal polyps depending on the iNOS expression at cellular and molecular levels.

In this study, we intended to histologically and genetically distinguish and assess iNOS expression in allergic and nonallergic NPs (NANP).

## 2. Material and Methods

### 2.1. Case Selection and Sample Collections

The current study is a prospective comparative study for evaluation of the expression of nitric oxide, by detecting the expression of iNOS in protein and mRNA in NPs. The tissue samples were collected at the Department of Otorhinolaryngology, Minia University Hospital, from July 2016 to February 2017. The study included 60 patients aged from 15 to 52 years old and of both sexes.

The patients were categorized into 3 groups. Each group included twenty patients as follows: *Group A*, *control group* (*concha bullosa group*), included 20 patients prepared for concha bullosa surgery (lateral lamellectomy) without any evidence of allergy. Biopsies were taken from the middle turbinate during operation and were considered as normal (control) nasal tissue. *Group B*, *study group* (*allergic nasal polyposis group*), included 20 allergic rhinitis patients with nasal polyposis prepared for functional endoscopic sinus surgery (FESS). *Group C*, *study group* (*nonallergic nasal polyposis group*), included 20 nonallergic rhinitis patients with nasal polyposis prepared for FESS surgery. Informed consent was taken from each patient after explanation of the procedure.

### 2.2. Ethics Statement

This study was carried out in accordance with the ethical guidelines of the 1975 Declaration of Helsinki and was approved by the medical ethics committee of the Faculty of Medicine—Minia University. Written informed consent was obtained from every participating patient.

### 2.3. Evaluation and Preparation of the Patients

#### 2.3.1. Clinical History

Detailed history of the ear, nose, and throat was taken from each patient (with special attention to nasal symptoms in the form of nasal obstruction, nasal discharge, headache, facial pain, smell disorders, snoring, allergic manifestations, history of bronchial asthma, and aspirin sensitivity) (Table 1).

The allergic nasal polyposis diagnosis depends mainly on family history focused on asthma, aspirin sensitivity, nasal polyposis, and atopy by usual response to the question, “Do any of your family members and/or relatives suffer from nasal polyposis, asthma, or atopy?” Allergic sensitization was evaluated by a skin prick test that was carried out and read according to guidelines. Results were considered positive if the major wheel diameter was 3 mm or greater than 16 of the commercial allergen panel (Stallergenes, Milan, Italy).

#### 2.3.2. Clinical Examination

Clinical examination of the nose by anterior rhiNOScopy was done, and nasal endoscopy (4 mm diameter, 0.30° nasal endoscope, KARL STORZ, Germany) was used in both nasal cavities to assess the NPs according to the Lund-Kennedy classification: 0: no polyposis; 1: polyposis restricted to the middle meatus; 2: occurrence of polyposis beyond the middle meatus (Table 1). Examination was done under local anesthesia by a ribbon gauze soaked with ephedrine : saline (1 : 1000)+xylocaine in both nasal cavities for 15 minutes.

#### 2.3.3. Computed Tomography (CT)

CT was performed for each patient in both coronal and axial views (Table 1).

### 2.4. Inclusion and Exclusion Criteria

Our study comprised 20 patients prepared for concha bullosa surgery without any evidence of allergy *Group A* (*concha bullosa group*) and 40 patients with bilateral NPs *Group B* (*allergic nasal polyposis group*) (20 patients) and *Group C* (*nonallergic nasal polyposis group*) (20 patients). The patients of both groups were (with grade 1 and 2 Lund-Kennedy classification) prepared for FESS as they showed no adequate improvement with proper medical treatment, which included systemic antibiotics, steroids, antihistamine, and decongestants for 2 weeks followed by topical steroids with or without systemic decongestants for more than 1 month. All our patients did not have previous nasal surgery with normal nasopharyngeal examination.

These patients were excluded from our study: those with unilateral nasal polyp who carried the possibility of malignancy, those with prior paranasal sinus surgery, those with bleeding tendency or hemoglobin less than 10 gm/dl (transient exclusion until correction of anemia (more than 12 gm/dl)), those who are smoking, and those who refused to undergo the procedure.

### 2.5. Surgery

All patients were admitted 24 hours before surgery; biopsies were taken during the surgery which was performed under general anesthesia. In *Group A*, *concha bullosa group*, the control group, specimens were taken (lateral lamellectomy). In *Group B* (*allergic nasal polyposis group*) and *Group C* (*nonallergic nasal polyposis group*), the study groups, specimens were taken from macroscopically observed polypoid areas from three sections: nasal cavity and maxillary and ethmoid sinuses (anterior and posterior).

### 2.6. Histological Study

Specimens were examined under light microscopy (×400 magnification); slides with polypoidal tissue were included in the study, and slides without polypoidal tissue (chronic inflammation only) were excluded. Specimens were examined at the Histology Department, Faculty of Medicine, Minia University, to detect the expression of iNOS.

### 2.7. The Paraffin Technique and Staining with Hematoxylin and Eosin (H&E) [12]

Specimens were immersed in 10% formal saline for fixation. Formalin was used for useful toughening effect and causes slight decrease of tissue size. After one or two days of fixation, the tissues were gradually immersed in alcohol (50%, 70%, and 90% and 3-time change of 95% alcohol). Then, the tissues were cleared using xylene. Soft paraffin (55-60°C) was successively changed 3 times. Lastly, the tissues were inserted in hard paraffin wax to get solid blocks holding the tissue samples.

Successive transverse pieces with thickness 5-6 *μ*m were prepared by a rotatory microtome. These sections were flattened by floating in a hot water bath and then putting on glass slides enclosed with albumin-glycerin. The sections were stained with hematoxylin and eosin (H&E) to be viewed by light microscopy for general histological study.

The dewaxed paraffin sections were put in hematoxylin stain for 2-20 minutes then washed well in running tap water for 2-3 minutes. The excess stain was removed by decolorizing in 5-10% HCl in 70% alcohol for a few seconds; then, the sections were put in 1% aqueous eosin for 1-3 minutes, and the surplus stain was put in water for washing. The sections were dehydrated by ethanol, cleared in xylene, and then mounted.

### 2.8. Immunohistochemical Study

Immunocytochemical staining was achieved via anti-Ki67 antibody (Sigma-Aldrich, Germany), anti-TGF beta antibody, and primary antibody mouse anti-human monoclonal antibody for iNOS (inducible nitric oxide synthase) from Lab Vision Laboratories (Thermo Fisher Scientific, USA). IHC was made on formalin-fixed, paraffin-embedded tissue according to the previous protocols [13, 14]. All samples were blindly tested, and positive cells were randomly counted in 20 selected fields in both lamina propria and epithelial compartments using an Olympus microscope (Olympus, Japan).

### 2.9. Quantitative Polymerase Chain Reaction RT-PCR

The RNA was isolated from the nasal tissues using the RNeasy FFPE Kit (Qiagen, Ltd., Crawley, United Kingdom) based on the manufacturer's guidelines. RNAse-free DNAse was added to RNA samples during the extraction procedure. cDNA was synthetized using the RETROscript kit (Applied Biosystems, Warrington, United Kingdom) based on the manufacturer's protocols. Briefly, reverse transcription (RT) was made using 2 ng total RNA from each studied group plus random primer in the presence of RNAse inhibitor (50°C; 1 hour). RT-PCR was performed using the synthetized cDNAs and SYBR green master mix (Applied Biosystems) and iNOS primers (forward: 5′CCTCAAGTCTTATTTCCTCAACGTT3′/reverse: 5′CCGATCAATCCAGGGTGCTA3′). *β*-Actin was used as the housekeeping gene (forward: 5′ATCCCCCAAAGTTCACAATG/reverse: 3′5′GTGGCTTTTAGGATGGCAAG 3′) (Metabion, Martinsried, Germany). All experiments were applied in triplicate. Results were recorded using Applied Biosystems™ 7500 Fast Real-Time PCR. Relative iNOS expression quantities were compared between the studied groups. The Ct were standardized against Ct of human *β*-actin.

### 2.10. Statistical Analysis

The data were expressed as the mean ± SD. Mann–Whitney *U* and Kruskal/Wallis tests were used in histological, immunohistologic, and genetical results, and *P* values < 0.05 are accepted as statistically significant.

## 3. Results

### 3.1. Patient Characteristic

The mean age ± SD of the studied cases was 31.5 ± 10 for Group A, 31.6 ± 13.2 for Group B, and 31.2 ± 10.2 for Group C (Table 1). Thirty-three cases were males while 27 cases were females. No significant associations were found between patients' age or sex and iNOS expression.

### 3.2. Histological Results

**Figure 1:**
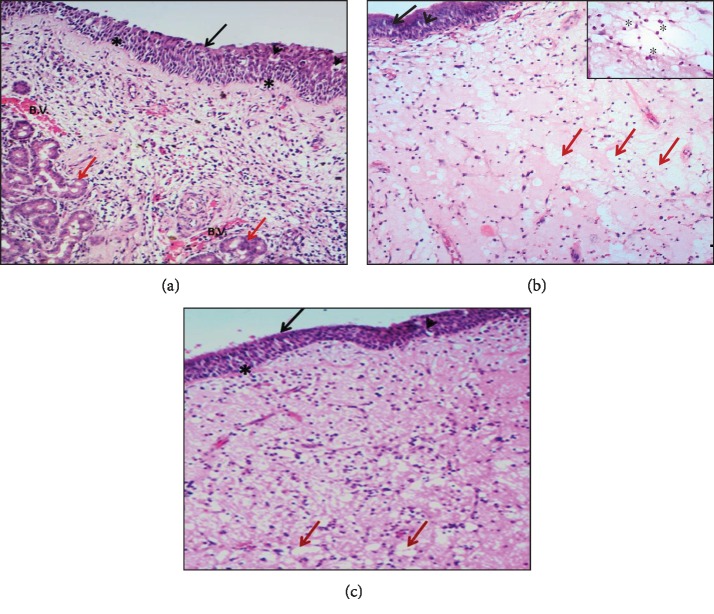
Photomicrograph of normal nasal mucosa (a), allergic nasal polyp (b), and nonallergic nasal polyp (c) stained with H&E ×400: (a) show pseudostratified columnar epithelium (arrow) with goblet cells (arrowhead) and thin basement membrane (asterisk). The stroma formed of loose C.T. tissue with blood vessels (B.V.) and seromucinous glands (red arrow). Notice that the cellular component of the stroma is mainly fibrous. (b) show pseudostratified columnar epithelium (arrow) with few goblet cells (arrowhead) and thin basement membrane (asterisk). Notice the high fluid content of the stroma (red arrow) with inflammatory cell infiltration and mainly eosinophils. The inset is a higher magnification of the stroma showing high content of eosinophils (asterisk). (c) show pseudostratified columnar epithelium (arrow) with few goblet cells (arrowhead) and thickened basement membrane (asterisk). Notice increase in the fluid content of the stroma (red arrow) with inflammatory cell infiltration and the absence of seromucinous glands and blood vessels.

The H&E stain experiment showed that Group A, control group (concha bullosa group), has normal nasal mucosa with pseudostratified ciliated epithelium with goblet cells resting on thin basal lamina and lamina propria of loose connective tissue containing multiple blood vessels. Some seromucinous glands could be detected in the section (Figure 1(a)). In Group B, study group (allergic nasal polyposis group), mucosa of the allergic nasal polyp showed an increase of the epithelial layers with squamous appearance with scarce goblet cells and increase in the thickness of basal lamina and lamina propria. Cell infiltration is mainly eosinophils (Figure 1(b)). For Group C, study group (nonallergic nasal polyposis group), in the mucosa of nonallergic nasal polyp, the epithelium showed less goblet cells and thickened basal lamina and lamina propria with inflammatory cell infiltration (eosinophils, lymphocytes, and plasma cells). Blood vessels and seromucinous glands could not be seen (Figure 1(c)).

### 3.3. Immunohistochemistry

**Figure 2:**
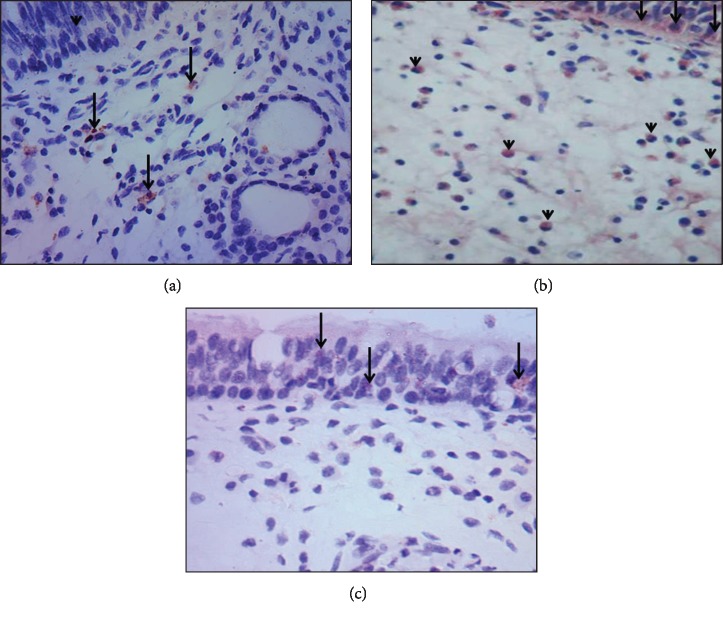
iNOS expression in normal (a), allergic nasal polyp (b), and nonallergic nasal polyps (c). Photomicrograph (×1000) of immune-histochemistry location of iNOS expression in normal nasal mucosa (a) showing few positive cells in the stroma (arrow). Notice the absence of the expression in the epithelial cells (arrowhead). Allergic nasal polyp (b) showing numerous positive cells in the epithelium (arrow) and stroma (arrowhead). Nonallergic nasal polyps (c) showing low expression in the epithelial cells (arrow) and nearly negative expression in the stroma.

**Figure 3:**
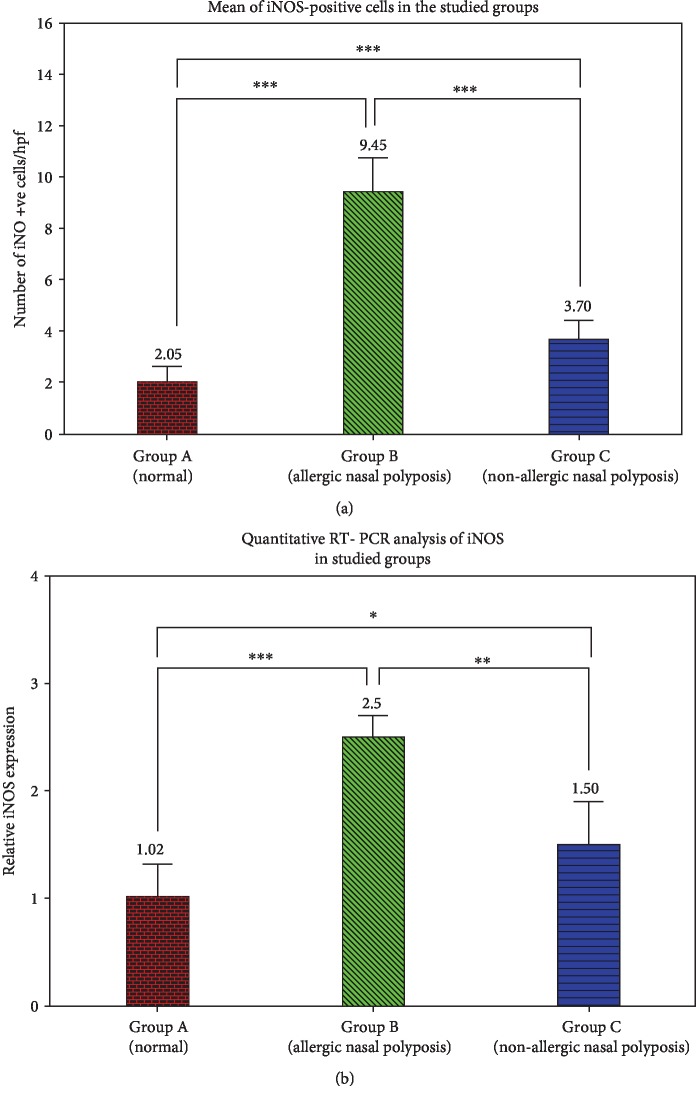
Comparison between groups according to the number of iNOS-positive cells (a) and quantitative RT-PCR analysis of the iNOS studied groups (b). Data expressed as mean ± SD of both positive iNOS cells in comparison to negative iNOS cells and mean ± SD of relative expression of iNOS in the studied groups. ^∗^ for *P* < 0.05 level, ^∗∗^ for *P* < 0.01 level, and ^∗∗∗^ for *P* < 0.001 level of significance.

Positive immunohistochemical staining of iNOS showed brown color in the cytoplasm. In Group A, control group (concha bullosa group), normal mucosa of the nose showed some positive cells in the stroma. Epithelial cells showed negative staining (Figure 2(a)). In Group B, study group (allergic nasal polyposis group), a high expression of iNOS could be detected in cytoplasm in both epithelial and inflammatory cells of the stroma, most probably eosinophils (Figure 2(b)). In Group C, study group (nonallergic nasal polyposis group), a weak cytoplasmic expression could be detected in some epithelial cells, but no positive cells could be detected in the stroma (Figure 2(c)). There was statistically significant difference between the 3 groups in regard to iNOS expression. Group B showed a higher expression when compared with both Group A (*P* value < 0.001) and Group C (*P* value < 0.001) while iNOS expression was higher in Group C than Group A (*P* value < 0.001) (Figure 3(a)).

**Figure 4:**
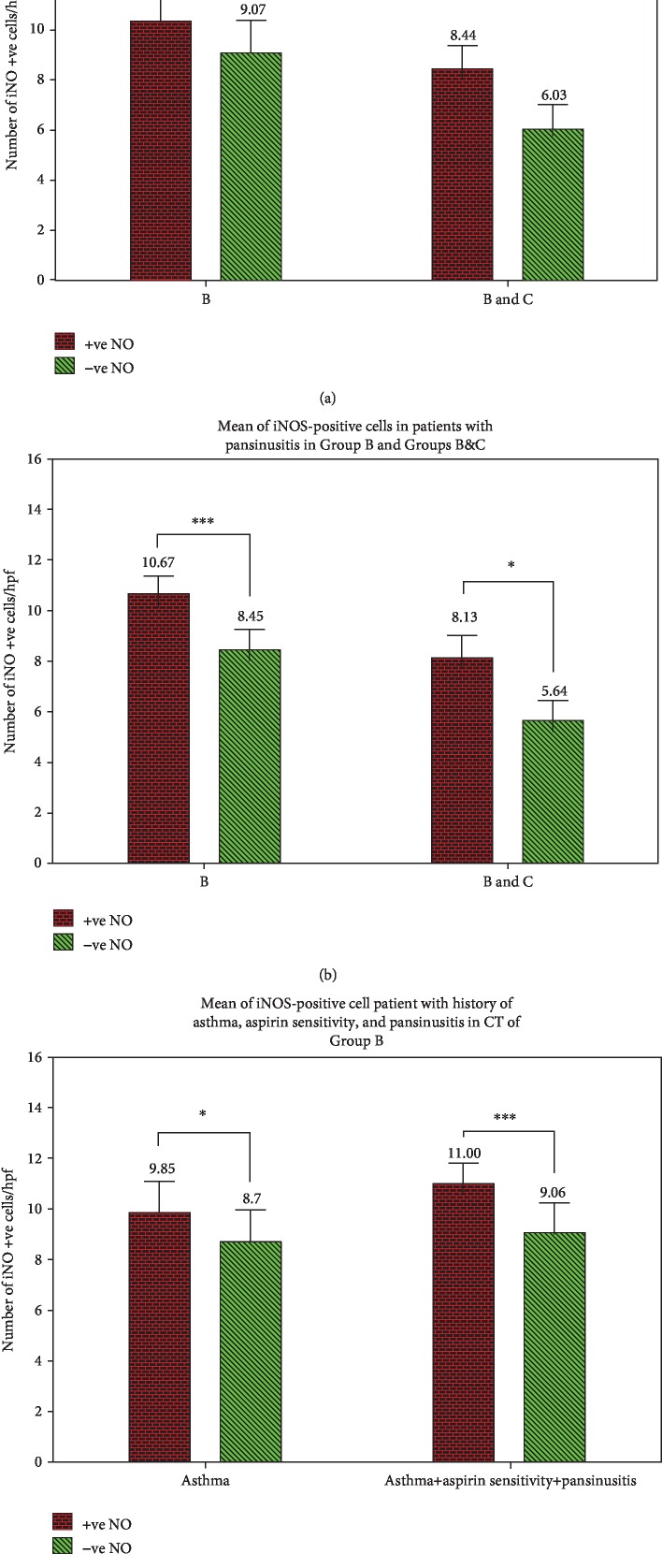
Mean of iNOS-positive cells in patients with history of aspirin sensitivity (a) in Groups B and B&C and pansinusitis (b) in Groups B and B&C and history of asthma with and without aspirin sensitivity and pansinusitis (c) in Group B. Data expressed as mean ± SD of positive iNOS cells in comparison to negative iNOS cells, ^∗^ for *P* < 0.05 and ^∗∗∗^ for *P* < 0.001 levels of significance. Group A (concha bullosa control group), Group B (allergic nasal polyposis group), and Group C (nonallergic nasal polyposis group).

iNOS expression was compared with other patients, but the results were statistically nonsignificant (*P* value was 0.584 in both Group B and Group C but 0.054 in Group B only) (Figure 4(a)). Patients with pansinusitis (in CT of the nose and paranasal sinuses) showed a higher expression when compared with other patients with partially affected sinuses in CT with statistically significant results (*P* value 0.042) (Figure 4(b)). Allergic patients with history of bronchial asthma had high iNOS expression but statistically nonsignificant (*P* value 0.074), but when those patients also had history of aspirin sensitivity and pansinusitis in CT, the results became significant (*P* value 0.007) (Figure 4(c)). The number of iNOS-positive cells in each patient of the studied groups is summarized in Table 2.

Allergic patients with history of bronchial asthma who are treated with steroids alone or in combination with bronchodilator had nonsignificantly low iNOS expression compared with bronchodilator cases.

### 3.4. iNOS mRNA Expression

Quantitative real-time PCR assays were performed on the isolated nasal tissue of studied groups. The expression of the iNOS gene was normalized by the expression of *β*-actin. iNOS was highly expressed in Group B (2.5-fold) compared to Groups A (1.02-fold; *P* < 0.001) and C (1.5-fold; *P* < 0.01) (Figure 3(b)). Our results also verified that the expression of iNOS gene is different between the B and C groups (*P* < 0.05; Figure 3(b)).

## 4. Discussion

NO is an imperative cellular signaling mediator, having an essential effect in several biological processes. NOS is highly expressed in activated cells produced under allergic or inflammatory situations [15]. iNOS is highly expressed as a response to inflammatory stimuli such as cytokines [16]. In allergic rhinitis, especially rhinitis, the epithelial cells of nasal mucosa are overexpressed with iNOS and produced high levels of NO resulting in increased the mucosal secretions [17]. We found that the expression of iNOS in NPs was much more than that of normal nasal mucosa (MT), and that of allergic patients was more than that of nonallergic ones. Similarly, NOS overexpressed in activated NP has been reported [18, 19]. Moreover, nasal NO levels are significantly elevated in healthy patients compared to chronic rhiNOSinusitis [20]. The reduction of NOS2 (iNOS) expression in patients with chronic sinusitis and NP has been described [21, 22]. Our results agree with a previous study; it was found that the numbers of positive cells (epithelial and stromal) of both iNOS and insulin-like growth factor-1 receptor were highly increased in NP compared to normal mucosa [23].

In addition, the previous study concluded that iNOS expression is upregulated in NP tissues and condensed mainly in the polyp epithelial layer [24]. However, the localization of iNOS in subepithelial tissue was not detected even in inflammatory cells [24]. Our results in allergic patient iNOS expression were detected in both epithelial and stromal layers. Inflammatory cells like macrophages and eosinophils also showed iNOS expression.

It has been established that the NOS isoforms [1–3] are overexpressed in all cells under the epithelium in NP more than normal MT [25]. They found that the percentage of mast cells, eosinophils, and macrophages that overexpressed NOS is higher than the percentage of neutrophils and T-cells [25]. Several reports have recognized that the expression and activity of NOS are high in NP [18, 19], in allergic rhinitis [17, 26, 27], and in aspirin-sensitive patients with asthma [28]. However, few studies have focused on the difference between NPs of allergic patients and nonallergic ones with regard to iNOS expression. Our results in this point agree with Ozcan et al. [10], who found that ACP (antrochoanal polyps) and ANP (allergic NPs) groups (epithelial and stromal) commonly exhibited moderate and severe overexpression of iNOS protein compared to NANP (nonallergic NPs) and inferior turbinate mucosa [29]. In agreement with Ozcan et al. [30], the low number of eosinophils, the high number of other inflammatory cells, the normal-appearing basement membrane, and intact and normal surface epithelium may reveal that the etiology of ACP (antrochoanal polyp) might arise from chronic inflammatory processes rather than allergy. The destruction of the endothelium may be considered as a further sign of chronic inflammation [30].

In our study, we noted that patients with extensive nasal polyposis (pansinusitis in CT) had higher iNOS expression than other patients with partially affected sinuses in CT with statistically significant results. In Group B (allergic nasal polyposis group), patients with history of asthma and aspirin sensitivity and had extensive nasal polyposis (pansinusitis in CT) showed the highest expression in our patients with statistically significant difference between them and other patients not having such a combination.

In our study, we noted that aspirin-sensitive patients showed higher iNOS expression when compared with other patients, but the results were statistically nonsignificant. In this point, we agree with Parikh et al. [28], who stated that polyps of aspirin-sensitive patients overexpressed with iNOS and proposed that NO has an impact in the aetiopathogenesis of NPs with aspirin-exacerbated respiratory disease (AERD) [28].

Patients with age over forty years have more frequency of the combination of aspirin sensitivity, asthma, and NPs. This classic condition is usually nonatopic. Children of parents without AERD are less susceptible to get chronic sinusitis with NPs than those children of parents with asthma, NPs, and aspirin sensitivity [31].

The iNOS mRNA expression has the same pattern of histological findings; the expression of iNOS was significantly increased in the ANP more than normal and NANP groups. A previous finding of the semiquantitative PCR results stated that iNOS expression was higher in the NP group compared with normal without coverage of a comparison of ANP and NANP [24].

Systemic and topical glucocorticoids [17, 32] and nasal decongestants [33] suppress the iNOS and decrease the level of nasal-derived NO. Many of our patients had used these medications prior to surgery especially local and systemic steroids and stopped them for a variable time before the surgery. This may explain the difference in iNOS expression between allergic and nonallergic patients (why some of our patients show significant iNOS expression while others do not). Such a problem should be put in consideration in future studies by adjustment of the dose and duration of steroid therapy and the period of stopping before surgery or biopsy taking.

Prostaglandin (PG) is produced by NO via activation of COX-II enzyme. PG is the main mediator of inflammation that includes edema and vascular permeability increase. Therefore, NOS inhibitors prevent the inflammatory reactions and have anti-inflammatory effects depending on their dose and the administration route [34]. In future studies, the effects of NOS inhibitors should be considered in order to evaluate the applied medical and surgical interventions on NP patients and to completely understand the NP pathogenesis and if they have a possible role in prevention of the recurrence. The small sample size in this study might affect the significant difference between studied groups. Large-scale study that includes more sample size with different types of allergy and treatment should be conducted to confirm the ability of iNOS expression to differentiate between ANP and NANP cases.

## 5. Conclusion

Our study showed that iNOS expression was much more in NPs than normal MT tissues in both epithelial and stromal layers. In addition, when the allergic group is compared with the nonallergic group, the NPs of the allergic group showed more iNOS expression in the epithelial layer with abundant eosinophilic and macrophage infiltration in the stromal layer. This quantitative pattern shows that iNOS and iNOS-derived NO play a role in the pathophysiology of NPs especially in patients with allergic rhinitis. Future studies should focus on the effect of steroid therapy on iNOS expression in NPs, the role of iNOS and NO in the recurrence of NPs, and the effect of NOS inhibitors in the medical treatment, preoperative preparation, or prevention of the recurrence of NPs.

## Figures and Tables

**Table 1:** Age, gender, clinical symptoms, clinical examination, and CT findings of the patients.

The symptoms/signs/findings	Number of positive cases		
	Group A *N* = 20	Group B *N* = 20	Group C *N* = 20
*Age (years)*			
Range	17-50	15-52	16-51
Mean ± SD	31.5 ± 10	31.6 ± 13.2	31.2 ± 10.2
Sex: male/female	9 (45%)/11 (55%)	12 (60%)/8 (40%)	12 (60%)/8 (40%)
*Clinical symptoms of the patients*			
Nasal obstruction	7	20	20
Nasal discharge	8	17	12
Headache	20	16	11
Facial pain	18	18	17
Smell disorders	0	19	15
Snoring	3	15	14
Allergic symptoms (itching, sneezing, and runny nose)	0	20	0
Bronchial asthma	0	13	2
Aspirin sensitivity	0	6	3
*Drug used*			
Bronchodilator		12	
Corticosteroids	0	3	0
Both		5	
*Clinical examination of the patients*			
Nasal mucosa (pale bluish in color)	0	20	0
Hypertrophied inferior turbinates	7	14	13
Concha bullosa	20	8	9
Nasal polyps (Lund-Kennedy class.)	0 = 20	0 = 0	0 = 0
	1 = 0	1 = 6	1 = 7
	2 = 0	2 = 14	2 = 13
*CT findings (affected paranasal sinuses in CT)*			
(i) Pansinusitis	0	9	6
(ii) Partially affected sinuses	0	11	14
(iii) Free sinuses	20	0	0

**Table 2:** Number of iNOS-positive cells in each patient of the studied groups.

Patient number	Number of iNOS-positive cells/hpf		
	Group A (concha bullosa control group) *N* = 20	Group B (allergic nasal polyposis group) *N* = 20	Group C (nonallergic nasal polyposis group) *N* = 20
1	2	7	5
2	2	10	3
3	1	9	3
4	2	9	4
5	3	11	3
6	3	9	4
7	2	8	4
8	1	9	4
9	3	9	4
10	2	8	5
11	2	9	3
12	1	10	4
13	2	9	3
14	2	7	3
15	2	12	3
16	3	11	3
17	2	11	5
18	2	10	3
19	1	10	4
20	2	11	4

## Data Availability

The authors stated that the data underlying the findings of this manuscript is available to share.
